# Association Between Sperm Metabolites and Field Fertility in Angus Bulls

**DOI:** 10.3390/metabo16050307

**Published:** 2026-04-30

**Authors:** Samantha R. Roberts, Sarah E. Moorey, Adella B. Lonas, Emma A. Hessock, Blessing A. Abiodun, Shawn R. Campagna, F. Neal Schrick, Saulo Menegatti Zoca

**Affiliations:** 1 Department of Animal Science, University of Tennessee Institute of Agriculture and AgResearch, 2506 River Drive, Knoxville, TN 37996, USA; 2 Department of Chemistry, University of Tennessee, 1420 Circle Dr., Knoxville, TN 37996, USA; 3 Biological and Small Molecule Mass Spectrometry Core, University of Tennessee, 1414 Circle Dr., Knoxville, TN 37996, USA

**Keywords:** sperm, bull fertility, metabolites, oxidative stress

## Abstract

Background/Objectives: Understanding the causes of bull subfertility and developing reliable diagnostic tools are critical to reducing economic losses caused by reproductive failure in beef cattle. Metabolomic analysis of sperm from bulls with diverging field fertility may provide insights on sperm metabolism that are associated with fertility. The objective was to determine metabolomic differences in sperm from bulls with differing field fertility. Methods: Angus bulls (*n* = 15) were classified based on a composite field fertility index (CFI). Frozen–thawed semen straws (*n* = 10 per bull) underwent a Percoll gradient sperm purification process. Metabolomic analysis was performed through ultra-high performance liquid chromatography coupled high resolution mass spectrometry at the University of Tennessee Biological and Small Molecule Mass Spectrometry Core. The general linear model (GLM) procedure of Statistical Analysis System (SAS) was used to evaluate linear and quadratic relationships between metabolites and CFI. Furthermore, the MIXED procedure was used to determine differences in metabolite abundance between the four highest and lowest fertility bulls. Significance was determined when *p* ≤ 0.05 and tendency was declared when *p* ≤ 0.10. Results: A total of 75 metabolites were detected. Quadratic relationships with fertility were observed for kynurenine, xanthine, and ophthalmate. Tricarballylic acid and creatinine showed a negative linear relationship with fertility. When differences in metabolite abundance were assessed between the four highest and lowest fertility bulls, *N*-acetylglutamate and *N*-acetylglutamine had greater abundance in low fertility bulls. Conclusions: Metabolites kynurenine, xanthine, ophthalmate, tricarballylic acid, and creatinine are potential fertility markers to identify subfertile bulls from a breeding population. These metabolites have promising future implications in the diagnosis and treatment of beef bull subfertility.

## 1. Introduction

The sustainability of cattle production systems relies on efficient reproduction and superior genetic merit. Artificial insemination (AI) is the most common assisted reproductive technology utilized in cattle, and its implementation can have strong positive impacts on both reproductive efficiency and genetic merit in beef and dairy herds [1]. Unfortunately, bovine pregnancy rates to a single AI with healthy females usually ranges from 30 to 60% [2,3,4]. While much focus is placed on the female, studies report that a large proportion of reproductive failure originates with the male [5,6].

The primary diagnostic tool for bovine male subfertility is a breeding soundness exam (BSE), which includes a physical examination (reproductive organs, structural soundness), scrotal circumference measurement, and a semen analysis (sperm morphology and motility) [7]. Approximately 20 to 25% of bulls fail a BSE and are classified as infertile or subfertile [8,9,10,11,12]. Moreover, bulls used for AI have gone through rigorous analyses and have passed additional quality control tests [13,14,15]. Nevertheless, semen evaluation of bulls only explains 50 to 60% of the variation in fertility observed in the field [16].

Sperm traits relevant for fertility have been classified into compensable and uncompensable traits as originally described by Saacke et al. [17]. Compensable traits related to the sperm’s inability to reach and fertilize the oocyte can be corrected for when the insemination dose is increased. Conversely, uncompensable traits associated with sperm capacity to block polyspermy and support embryo development post fertilization cannot be corrected by increased insemination dosage [17,18]. Recently, it was reported that the insemination dose used commercially is sufficient to overcome the compensable component of semen quality; however, differences in fertility between bulls was still present [19]. Thus, the development of new fertility markers associated with uncompensable traits that impact field fertility are necessary.

Metabolomic analyses of sperm cells allow one to more holistically assess the intracellular conditions of sperm and begin to decipher the causes of uncompensable traits that are detrimental to cattle fertility. Metabolomics is the study of the products of metabolism, including small, low-molecular weight metabolites [20]. Initial metabolome studies with dairy bulls have provided promising outlooks on the potential to discriminate bulls of high and low field fertility based on metabolome profiles. While most metabolome studies focus on seminal plasma [21,22], limited studies have focused on the sperm cell itself. Menezes et al. [23] reported that there were 22 differentially regulated metabolites in sperm cells from fresh ejaculate of Holstein bulls of high or low fertility. Most metabolites related to fertility classification were organic acids and their derivatives or fatty acids and their conjugates, suggesting that bull spermatozoa have an active energy metabolism and that high fertility bulls had a greater abundance of metabolites that are utilized for energy [23]. Furthermore, analysis of purified sperm samples of high and low fertility Holstein Friesian bulls revealed 44 and 35 unique metabolites associated with high and low fertility, respectively, along with 56 metabolites common to both groups [24]. It was reported that the high fertility bulls had a higher energy status in the spermatozoa and that metabolites present in this classification can aid sperm motility [24]. In addition, it has been demonstrated at multiple levels (transcriptomic, proteomic, and metabolomic) that sperm metabolism is likely involved in the regulation of fertility in bulls [25].

To the best of the authors’ knowledge, all research in this high impact area has focused on dairy bulls, leaving a significant knowledge gap regarding beef bulls. Additional accounts of metabolomic differences between sperm cells of beef bulls with differing field fertility are required to fully understand the metabolomic signature of fertility in male bovine and determine if the limited reports in dairy are translatable to beef bulls. Metabolomic insight into subfertile sperm may become a useful biomarker for bull fertility or be used to develop fertility restoring treatments for subfertile bulls and potentially humans. The objective of the current study was to profile the sperm metabolome of Angus beef bulls with variable fertility and determine metabolomic differences in sperm related to field fertility. The hypothesis was that sperm metabolome profiles associated with energy production and oxidative balance would differ based on field fertility of bull [23,24,25,26].

## 2. Materials and Methods

### 2.1. Animal Use Statement

No live animals were used for this project. Semen and fertility data were provided by Select Sires Inc. (Plain City, OH, USA).

### 2.2. Experimental Design

**Figure 1:**
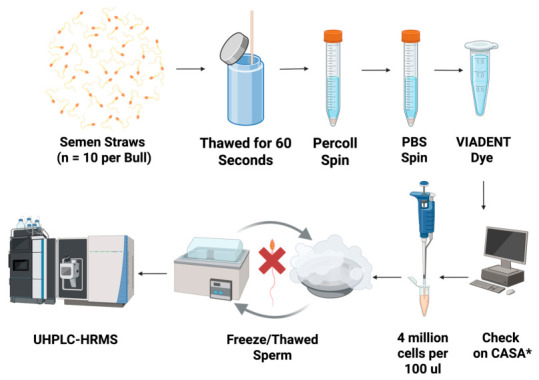
Semen straws (*n* = 10 straws of each bull from a single ejaculate) were thawed in a water bath at 37 °C for 60 s. A 45–90% percoll protocol was performed to separate live sperm from non-motile sperm, dead sperm, and extender. Sperm quality was assessed on a CASA using VIADENT dye (10 µg/mL; IVOS II, Hamilton Thorne, Beverly, MA, USA). A 100 µL aliquot of each sample containing 4 million sperm underwent 5 rounds of freezing–thawing in liquid nitrogen and water bath (37 °C) for cell lyses. Sperm metabolome profiles were generated using UHPLC-HRMS at the University of Tennessee Biological and Small Molecule Mass Spectrometry Core. Created with BioRender.com. * CASA = Computer assisted sperm analysis.

Frozen artificial insemination straws containing semen from Angus beef bulls of variable field fertility (*n* = 15 bulls; composite fertility index (CFI) = −7.3 to 3.3) underwent a Percoll protocol to separate live sperm from extender as well as non-motile and dead sperm cells. Live sperm were lysed via five rounds of freeze–thaw (liquid nitrogen—water bath at 37 °C) and were submitted to ultra-high performance liquid chromatography coupled with high resolution mass spectrometry (UHPLC-HRMS) in aliquots of equal volume and cell number. Relationships between sperm metabolome profiles and bull fertility were assessed based on a combination of regression analyses of field fertility and metabolite abundance of all bulls and comparing mean metabolite abundance between the four highest and lowest fertility bulls (Figure 1).

### 2.3. Bull Fertility Classification

Bulls were classified based on a field fertility index (CFI) from Select Sires Inc. where zero represented average fertility, a positive number represented above average, and a negative number represented below average fertility bulls. To calculate CFI, Select Sires Inc. used two datasets and calculated a combined weighted deviation based on number of inseminations. Data were retrieved from a nationwide dataset (AgriTech Analytics, Visalia, CA, USA; ATA) and the Select Sires Inc. internal data set. Duplicates between ATA and Select Sires Inc. database were minimal to non-existent. Sire Conception Rates (SCR) data were not included in CFI calculation because it would generate duplicates. All data were generated from Angus bulls used in Holstein dairy herds in the USA. To be considered for the present study, Angus bulls with available CFI from Select Sires Inc. were required to have a minimum of 1000 inseminations (*n* all = 15, range = 1060 to 32,273; four highest = 4739 ± 1715; four lowest = 3020 ± 1166). The CFI of all bulls ranged from −7.3 to 3, and the average CFI of the 4 highest (High: 2.30 ± 0.35) and lowest (Low: −5.25 ± 0.93) fertility bulls differed (*p* < 0.0001). The bulls’ ages at collection ranged from 1.72 to 7.92 years old. Sample size in the current study was limited to 15 bulls because of limited availability of semen from bulls with low CFI.

### 2.4. Semen Processing

The purification process used in this study was an attempt to eliminate non-motile and dead sperm that would not reach the site of fertilization and increase the chances of detecting true biological differences relevant to uncompensable traits related to bull fertility. Semen samples underwent a Percoll protocol (45%/90% layered Percoll gradient) for sperm purification and removal of semen extender (adapted from [27]). Semen was processed by one of two technicians each day. Frozen straws of bull semen (*n* = 10 per bull, collected from a single ejaculate) were thawed in a water bath at 37 °C for 60 s. A preliminary study was performed and determined that 10 straws were required to confidently collect the required sperm cell numbers, post Percoll purification, for metabolomics analysis on all bulls. Content of straws were combined in a 15 mL conical tube. Semen was then carefully layered on top of the Percoll gradient and centrifuged for 17 min at 760× *g*. The resulting pellet of sperm cells was then transferred into a new 15 mL conical containing 10 mL of Phosphate-Buffered Saline (PBS; pH 7.4) and centrifuged for 10 min at 300× *g*. After Percoll processing and washing procedures, the sperm pellet was diluted with PBS before being analyzed on the Computer-Assisted Sperm Analysis system (CASA; IVOS II, Hamilton Thorn, Beverly, MA, USA) to assess Percoll protocol efficiency through sperm motility, viability, and concentration. The VIADENT fluorescence option was used on the CASA to assess sperm viability using 10 µL of sperm samples and 10 µL of VIADENT stain (10 µg/mL).

After retrieving sperm concentration from the CASA results, four million cells were aliquoted for metabolomics and diluted to a final volume of 100 µL with PBS (40 × 10^6^ sperm/mL). Sperm characteristics for all bulls with CFI > 0 (*n* = 8) and CFI < 0 (*n* = 7) were not different for total motility (77.1% ± 5.6% vs. 78.5% ± 5.5% for >0 and <0, respectively; *p* = 0.70), progressive motility (60.1% ± 5.7% vs. 65.9% ± 5.5% for >0 and <0, respectively; *p* = 0.21, and viability (95.3% ± 1.3% vs. 93.9% ± 1.8% for >0 and <0, respectively; *p* = 0.51). Further, sperm characteristics for the four highest and four lowest fertility bulls did not differ (*p* ≥ 0.45) for total motility, progressive motility, and viability (Table 1). Thus, indicating that the sperm submitted for metabolomic analyses were not different between fertility classifications.

### 2.5. Metabolome Profiling

After the Percoll protocol was performed, 100 µL aliquots of each sample (40 × 10^6^ sperm/mL) underwent five rounds of freezing (liquid nitrogen) and thawing (water abth at 37 °C) to lyse sperm cells and stop metabolism. Samples were then snap frozen in liquid nitrogen and stored at −80 °C until further analyses. All sperm samples were processed at the University of Tennessee Biological and Small Molecule Mass Spectrometry Core (RRID: SCR_021368) for UHPLC-HRMS similarly to Horn et al. [28]. Briefly, metabolites were extracted from the samples using a 20:40:40 water/methanol/acetonitrile solution with 0.1 M formic acid [29,30]. Extraction solvent (1.3 mL) was added to the samples and vortexed, then chilled for 20 min at −20 °C. These tubes then underwent centrifugation (2500× *g* for 5 min at 4 °C) until pellets formed. Debris was removed from the liquid suspension via filtration, and the supernatant was transferred to a fresh set of microcentrifuge tubes. Fresh extraction solvent of 200 uL was added to the remaining extration pellets, and a pipette tip was used to suspend the material. The resuspending pellets were extracted as stated above (vortexed, chilled, and centrifuged). The second aliquot of supernatant was removed and combined with the first. Nitrogen gas was used to dry samples, which were then resuspended in 300 uL MilliQ water and transferred to autosampler vials. Metabolites for each sample were separated on a Synergi Hydro RP, 2.5 μm, 100 mm × 2.0 mm column (Phenomenex, Torrance, CA, USA) fitted to an Exactive^TM^ Plus Orbitrap mass spectrometer (Thermo Fisher Scientific, Waltham, MA, USA), which was maintained at 25 °C. The solvents for the mobile phase for elution were 97:3 methanol to water (*v*:*v*) with added tributylamine (11 mM) and acetic acid (5 mM) (Solvent A), and 100% methanol (Solvent B). The solvents for the 25 min eluction gradients are as follows: 0 to 5 min—100% Solvent A, 0% Solvent B; from 5 to 13 min—80% Solvent A, 20% Solvent B; from 13 to 15.5 min—45% Solvent A, 55% Solvent B; from 15.5 to 19 min—5% Solvent A, 95% Solvent B; from 19 to 25 min—100% Solvent A, 0% Solvent B. A constant flow rate of 0.2 mL/min was used for the entire separation. An Exactive Plus Orbitrap mass spectrometer (Thermo Fisher Scientific, Waltham, MA, USA) fitted with electrospray ionization was used to detect metabolites in negative ion mode via an untargeted analysis from a 10 μL injection of the resuspended combined extracts. Masses were scanned from 72 to 1000 *m*/*z* with a resolution of 140,000 and acquisition gain control of 3 × 10^6^ [31].

The data were saved as Xcalibur (.RAW) format and then converted to open source mzML format (msconvert software version 3.0; ProteoWizard package version 3.0). The Elucidata group metabolomic analysis and visualization engine (El-MAVEN) (v.0.12.0) was used to preprocess the samples, which included nonlinear retention time matching, peak picking, and visualization of extracted ion chromaotgrams [32]. Peak shape, exact mass (±5 ppm), retention time (±2 min), and a signal-to-noise ratio of 3:1 were used to manually validate metabolite detection and identify peaks from our standard metabolite library that has been validated as previously reported [30,32]. Tables of processed peak areas that were generated from El-MAVEN were used for downstream statistical analysis.

### 2.6. Statistical Analyses and Bioinformatics

Statistical analyses were performed in Statistical Analysis System (SAS) 9.4 (SAS Institute Inc., Cary, NC, USA). For each metabolite, datapoints greater than 2.5 standard deviations from the mean were considered outliers and were removed from statistical analyses. Normality was assessed using Shapiro–Wilk test, and metabolites were log transformed if required to reach normality (CDP-ethanolamine; Abscisate; 1-Methyladenosine; 2-Aminoadipate; AMP/dGMP; Aconitate; Adenosine; CMP; D-Glyceraldehdye 3-phosphate; Glucosamine; Glutamate; Glutathione disulfide; Glycerone phosphate; Hypoxanthine; IMP; *N*-Acetylglucosamine; *N*-Acetylglucosamine 1/6-phosphate; *N*-Acetylglutamine; Trehalose/Sucrose; UMP; Uridine; Xanthine; Xylitol). MetaboAnalyst 6.0 [33] was used to perform sparse partial least squares discriminant analysis (sPLSDA) of metabolome data of the four highest fertility and four lowest fertility bulls. The general linear model (GLM) procedure of SAS was used to evaluate linear and quadratic relationships between metabolites and CFI in all bulls where metabolite was used as fixed effect and CFI as dependent variable. After linear and quadratic analyses, the four highest and the four lowest fertility bulls were used to detect differences in metabolite abundance between CFI classification. The MIXED procedure was used with the model including the dependent variable of metabolite and the fixed effect of classification. For both analyses, a random statement was used to control for the date of semen processing and technician. Significance was determined when *p* < 0.05 and tendency was declared when *p* ≤ 0.10.

## 3. Results

### 3.1. Overview of Metabolome Data

There were 75 metabolites identified in sperm samples from Angus beef bulls, and all metabolites were present in both fertility groups. Metabolites were predominantly amino acids and organic acids.

### 3.2. Relationship Between CFI and Sperm Metabolite Abundance

**Figure 2:**
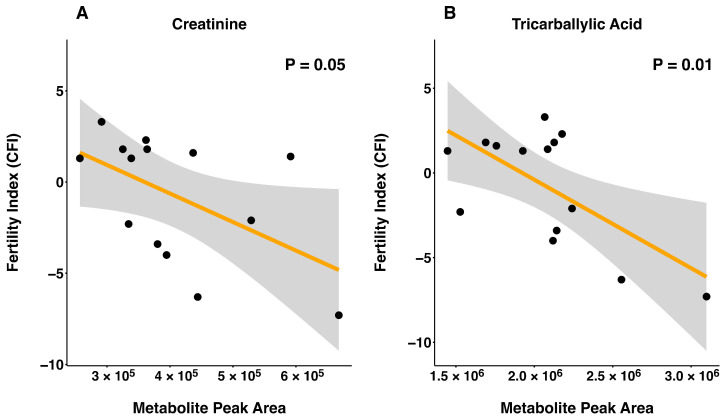
Linear relationships between metabolites and field fertility index (CFI) where zero represents average fertility, positive values represent increased fertility from average and negative values represent decreased fertility from average. Linear relationship for creatinine (**A**) and tricarballylic acid (**B**) with CFI. The *X* axis denotes peak area, indicating abundance of each metabolite and the *Y* axis denotes CFI. Black circles represent individual sample observations. The orange line indicates the fitted linear regression line. The gray shaded region represents the 95% confidence interval surrounding the regression estimate.

**Figure 3:**
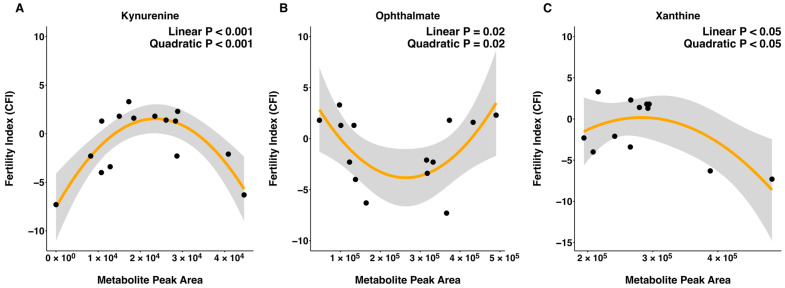
Quadratic relationships between metabolites and field fertility index (CFI) where zero represents average fertility, positive values represent increased fertility from average and negative values represent decreased fertility from the average. Quadratic relationship for CFI with kynurenine (**A**), ophthalmate (**B**), and xanthine (**C**). Model *p* ≤ 0.05. The *X* axis denotes peak area, indicating abundance of each metabolite and the *Y* axis denotes CFI. Black circles represent individual sample observations. The orange line indicates the fitted quadratic regression line. The gray shaded region represents the 95% confidence interval surrounding the regression estimate.

Sperm metabolite abundance and CFI were evaluated for linear and quadratic relationships using all bulls (Table S1). There was a linear relationship between CFI and metabolite abundance detected for tricarballylic acid and creatinine, where both metabolites were negatively related to CFI (*p* ≤ 0.05; Figure 2). Furthermore, quadratic relationships between CFI and the abundance of kynurenine (*p* < 0.001), xanthine (*p* = 0.03), and ophthalmate (*p* = 0.02) were detected (Figure 3).

### 3.3. Differential Metabolome Profiles of Sperm from Bulls Classified as High and Low Fertility

**Figure 4:**
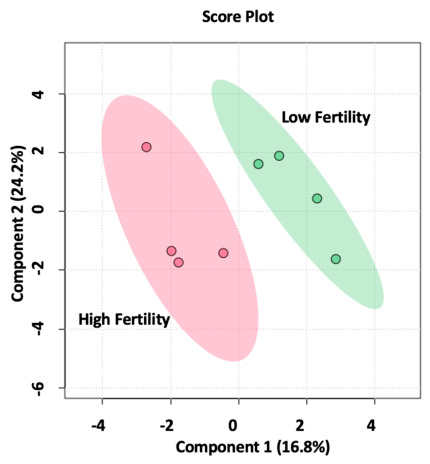
The sPLSDA plot of the four highest and lowest fertility Angus bulls indicated that samples from the four highest and lowest CFI bulls clustered independently based on metabolome profiles. Red and green dots represent individual samples from the four highest and lowest fertility bulls, respectively. Red and green shaded ellipses represent the 95% confidence area for the graphical location of high or low fertility samples, respectively.

The sPLSDA plot showed that samples clustered based on metabolite abundance in the four highest and lowest fertility bulls (Figure 4). Analysis was set to use the ten metabolites that most explained the variation among samples, and these metabolites were: *N*-acetylglutamate, pyroglutamic acid, *N*-acetylglutamine, creatinine, phosphoenolpyruvate, tyrosine, tricarballylic acid, aspartate, 3-phosphoglycerate, and creatine.

**Figure 5:**
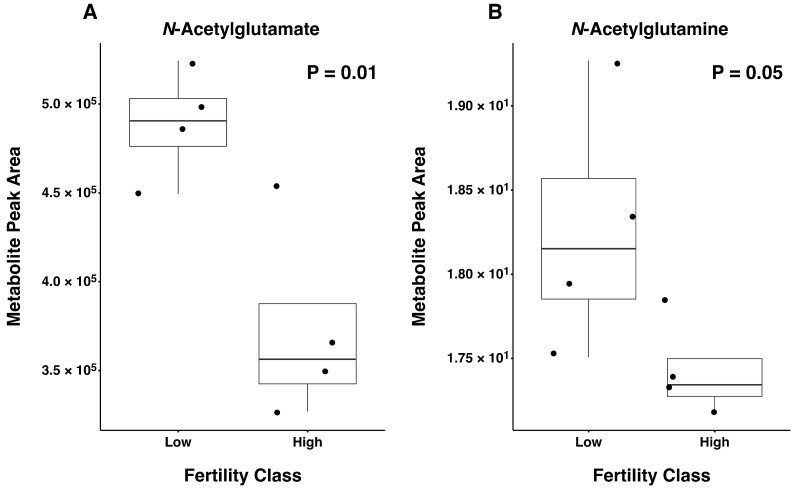
Boxplot with mean, quartiles, 95% confidence interval and datapoints (solid dot) for the four highest fertility (High) and lowest fertility (Low) Angus bulls for *N*-Acetylglutamate (**A**) and *N*-Acetylglutamine (**B**). The *Y* axis denotes peak area, indicating abundance, of each metabolite.

Next, differences in metabolite abundance were assessed between the four highest and four lowest CFI bulls (Table S2). Metabolites *N*-acetyl-glutamate (*p* = 0.01) and *N*-acetyl-glutamine (*p* = 0.05) had greater abundance in low fertility bulls (Figure 5). Similarly, pyroglutamic acid (*p* = 0.06; high = 52,787,813 ± 4,303,556; low = 66,427,748 ± 4,303,556; fold change = −1.26) tended to be more abundant in low fertility bulls, whereas 3-phosphoglycerate abundance tended to be greater in high versus low fertility bulls (*p* = 0.08; high = 1,141,332 ± 482,825; low = 221,929 ± 474,188; fold change = 4.46).

## 4. Discussion

Since AI is the most common assisted reproductive technology in cattle, it is important to understand the potential causes and treatments for bull subfertility [1]. Semen evaluations may explain up to 60% of variation in bull fertility [16]. Furthermore, an ejaculate or insemination dose can be divided into three populations (1) a population of sperm that will not initiate fertilization (compensable), (2) a population that will initiate fertilization but will not sustain embryo development (uncompensable), and (3) a fully competent sperm that can initiate fertilization and support embryo development. The relationship and proportion of these three populations will determine the fertility of an ejaculate, or insemination dose, or even a sire [34]. Each ejaculate, or insemination dose, must have “enough of” all traits for it be considered of high fertility. Nevertheless, what is enough for each trait is still unknown; therefore, the prediction of male fertility remains a great challenge [35]. Thus, there is a need to develop new fertility markers that can improve the fertility prediction of males. It is unlikely, however, that a single measure will explain most of the fertility variation, but rather a combination of multiple traits will improve our ability to predict male fertility. Metabolomic analysis allows for a holistic approach to sperm metabolism where multiple metabolites that impact different sperm functions can be explored simultaneously in the same population. Several studies have suggested that sperm metabolism may play a role in sperm longevity [26] and fertility [23,24,25]. A limited number of studies have been published associating metabolomic analyses with bulls’ fertility and most of those studies have been done in dairy bulls; thus, the current study aimed to understand the relationship of metabolites with beef bulls’ fertility.

The present study highlighted linear and quadratic relationships between sperm metabolites and fertility. The importance of quadratic relationships in complex traits such as fertility should not be overlooked. Disruption of biological function can be generated by an excess of or absence of specific compounds such as minerals, proteins, hormones, cytokines, and metabolites, to name a few. Differences in sperm metabolome profiles related to field fertility primarily highlighted processes involved in oxidative balance and energy metabolism that may be dysregulated in Angus bulls of subfertility.

Sperm cells have large energetic requirements of motility, capacitation, and fertilization [36,37]. Therefore, appropriate function of metabolic pathways related to ATP production are essential for optimal sperm fertility. Based on the results of the current study, it appears that ATP production may be reduced in sperm cells of Angus bulls with low fertility. Sperm cells primarily produce ATP through the glycolytic pathway and oxidative phosphorylation. Production of ATP for sperm motility is essential for optimal fertility, and most ATP used for motility is produced by the sperm’s midpiece using oxidative phosphorylation [38]. It has also been hypothesized that energy production shifts from a predominantly glycolytic manner in the epididymis to a more oxidative phosphorylation-based manner in ejaculated sperm, which was associated with increased sperm motility and decreased sperm longevity [26]. This change in energy production, from less efficient (glycolysis) to more efficient (oxidative phosphorylation) is necessary as sperm continues to progress towards fertilization; sperm hyperactivation and capacitation increases consumption of ATP and consequently the requirement for ATP production [39]. Failure of sperm cells to make this metabolic transition and produce required ATP for such processes could substantially reduce the fertilizing ability of an ejaculate.

Related to oxidative phosphorylation, tricarballylic acid had a negative linear relationship with field fertility. Interestingly, this metabolite has been known to interfere with TCA cycle function since it has an inhibitory effect on the enzyme aconitase [40]. Further, tricarballylic acid has a very similar molecular structure to citric acid; however, it lacks a hydroxyl group which is essential for aconitase to convert citrate to isocitrate; therefore, it effectively binds aconitase but prevents TCA cycle progression. Lack of metabolic progression through the TCA cycle would reduce the availability of molecules such as NADH that serve as essential cofactors in oxidative phosphorylation. Although bull sperm relies primarily on oxidative phosphorylation to produce ATP in the mitochondria (midpiece) [41], the sperm head and tail produce ATP primarily via glycolysis for capacitation and the acrosome reaction [42]. The metabolite 3-phosphoglycerate, which tended to be more abundant in the four highest fertility bulls compared to the four lowest fertility bulls, is the product of the seventh step of glycolysis, which produces two ATP molecules [43]. Although the current study did not detect pyruvate, whose reaction produces the additional two ATP of glycolysis, in the sperm cells, one can speculate that greater abundance of 3-phosphoglycerate may be indicative of superior glycolytic activity and ATP production in the sperm of high versus low fertility bulls. One study in pigs demonstrated specific implications of energetic pathway activity on fertility milestones in vitro, in which oxidative phosphorylation was related to fertilization rate; however, further development of preimplantation embryos was strongly impacted by sperm glycolysis [44]. Impaired glycolytic activity in subfertile bulls may therefore explain aspects of uncompensable traits and provide opportunity for future studies to better understand and improve energetic ability in such a population of animals. Regardless, it is important to note that ATP was not measured in the current study, and future studies to measure glycolytic activity or TCA cycle progression in subfertile versus highly fertile bulls are necessary to test hypotheses generated by these results. One limitation of the current study is that citrate was not discriminated from isocitrate in metabolite identification and α-ketoglutarate, the next metabolite in the TCA cycle, was not identified in the samples. Therefore, statements and hypothesis regarding impacts of tricarballylic acid on TCA cycles progression are speculative and should be tested in future studies.

Although creatine metabolism is not as readily associated with energy production in all cell types, it was suggested that the creatine kinase/phosphorylcreatine/creatine system was extremely important for ATP production in high energy cells, such as spermatozoa. This happens through phosphorylcreatine and creatine interconversion which provides a shuttle system for the production and consumption of ATP [45]. Creatinine, which was negatively related to CFI, is the final product of creatine and phosphorylcreatine metabolism and is produced by the degradation of either creatine or phosphorylcreatine. Abundance of creatine was not related to field fertility in this dataset, and phosphorylcreatine was not detected in the samples. Therefore, increasing abundance of creatinine may indicate reduced bioavailability of phosphorylcreatine in the sperm cells of bulls of reduced fertility, thereby leading to reduced efficacy of the phosphorylcreatine/creatine shuttle for energy production and utilization. Although no reports of creatinine’s relationship with male fertility were reported, it is interesting that supplementation of creatine to in vitro fertilization media, which could have promoted ATP production through the phosphorylcreatine/creatine shuttle, improved mouse sperm motility and capacitation [46].

Energy production and metabolism, especially through oxidative phosphorylation, produces reactive oxygen species (ROS) as a by-product. In moderate or physiological conditions, ROS can have physiological functions associated with sperm capacitation and hyperactivation that culminate in protein modification associated with calcium uptake and an increase in cAMP that are essential for fertility [47,48]. Nevertheless, increased concentrations of ROS can have deleterious effects associated with oxidation of proteins, lipids and DNA, that lead to genomic and physiological disfunctions [49] which highlights the importance of an oxidative balance for normal sperm function [50]. The increased metabolism of sperm through increased motility post-ejaculation is a desirable characteristic [26] which culminates with increased ROS production. Sperm cells contain little cytoplasm which results in them having a deficiency in antioxidant enzymes to protect from oxidative stress unlike other cell types [51]. The control of the abundance of ROS is largely regulated through antioxidant molecules that are necessary to maintain an oxidative balance without reaching levels of oxidative stress [52,53]. Thus, it is important that the sperm carries increased levels of antioxidants to prevent lipid peroxidation and DNA damage.

Kynurenine had a quadratic relationship to fertility, where low fertility bulls had lower or greater abundance of kynurenine compared to high fertility animals. The kynurenine cycle produces energy for cells, and yields both kynurenic acid and NADP+, which are both endogenous antioxidants required for oxidative balance under normal physiological conditions [54,55,56]. Furthermore, a study in mice demonstrated that knockout of indoleamine 2,3-dioxygenase, which catalyzes the rate limiting step of the kynurenine cycle, impacted immune tolerance and was associated with abnormal sperm quality [57]. The kynurenine cycle is also the major source for tryptophan degradation [55], and tryptophan can also serve as an antioxidant at normal intracellular concentrations. When tryptophan was supplemented at different doses in buffalo tris citric acid extender, there was an improvement in semen quality parameters post-thaw, including: progressive motility, plasma membrane integrity, mitochondrial membrane potential, acrosome membrane integrity, and DNA integrity, demonstrating tryptophan’s role as an essential antioxidant to help improve sperm quality at these various cellular levels [58]. Another study reported that the kynurenine pathway can expedite apoptosis and upregulate NADPH oxidase-derived ROS in endothelial cells at higher-than-normal intracellular concentrations [59]. Given the results of the current study, we speculate that in low fertility bulls with low kynurenine there may be a lack of antioxidants since kynurenine would not be converted to NADP(+) [56]; however, when kynurenine is in excess it may be working to expedite apoptosis and upregulate NADPH oxidase-derived ROS [59].

Xanthine had a quadratic relationship with bull fertility, with the two lowest fertility bulls having greater xanthine abundance than all high fertility and the remainder of low fertility bulls. Xanthine derivatives have been utilized in assisted reproduction to improve sperm motility in humans and mice [60]. Most xanthine related research in sperm, however, focuses on its conversion from hypoxanthine by xanthine oxidase to yield superoxide and hydrogen peroxide. When xanthine oxidase was added to human sperm (0.04 U) at 0 and 15 min timepoint incubations to increase ROS, fertility was decreased. At a lower dose (0.0287 U ml-1) in the same timepoint incubations, however, xanthine oxidase did not influence sperm motility but decreased sperm–oocyte fusion [61]. Given the results of the current study, excessive bioavailability of xanthine may be playing a role in increasing ROS to a high concentration that has been reported to increase lipid peroxidation and DNA damage [61,62], thus, causing lower fertility in some bulls.

Two additional metabolites that were associated, or tended to be associated, with bull fertility in our dataset are related to glutathione production (ophthalmate and pyroglutamic acid). It has been suggested that glutathione plays a pivotal role in guarding sperm from oxidative stress [63]. Meister [64] states that when cells are deprived of glutathione, they are more prone to suffer from severe oxidative damage that is associated with degeneration of the mitochondria. Ophthalmate had a quadratic relationship with bull fertility and is an analog of glutathione. It has been shown to be a potential biomarker for depletion of glutathione in rabbits, rats, and humans [65,66,67]. A study in mouse liver cells suggested that ophthalmate could potentially be a biomarker for oxidative stress since it is synthesized by the same enzymes [67]. Glutathione exerts negative feedback on the enzyme glutamyl-cysteine-synthetase; but during times of oxidative stress, this negative feedback disappears due to depletion of cysteine that results in production of more ophthalmate [68]. Due to the quadratic nature of ophthalmate’s relationship with fertility, high fertility bulls had either a greater or lower concentration of ophthalmate, with lower fertility bulls being intermediary. Accordingly, one might speculate that the high fertility bulls at the greater ophthalmate abundance are actively combatting oxidative stress and the sperm of high fertility bulls at low ophthalmate abundance were not under oxidative stress. Pyroglutamic acid tended to be more abundant in the sperm cells of low fertility bulls. Studies have demonstrated an inverse relationship between glutathione and pyroglutamic acid; when glutathione was decreased, pyroglutamic acid was increased [69,70]. Pyroglutamic acid is an intermediate metabolite in the glutathione cycle [71]. It has been reported that pyroglutamic acid abundance was elevated in the blood plasma of infertile men compared to fertile, and it was hypothesized that the increase in pyroglutamic acid was due to elevated ROS [72].

In addition, *N*-acetylglutamine was more abundant in the four lowest compared to the highest fertility bulls. A recent study reported that *N*-acetylglutamine was three times higher in Gaoqing bulls with low versus high motility [73]. To our knowledge, the current study is the first to report a link between *N*-acetylglutamine and fertility in beef bulls. *N*-acetylglutamate was also more abundant in the four lowest fertility bulls compared to the four highest fertility bulls. *N*-acetylglutamate is formed by *N*-acetylglutamate synthase from glutamate and acetyl-CoA, and this activates the enzyme carbamoyl phosphate synthetase-1 that starts the urea cycle [74]. This is the first, to our knowledge, that *N*-acetylglutamate has been associated with fertility. Further studies of these two metabolites are needed to be investigate their impacts on beef bull fertility. It is interesting that the metabolites identified as linearly related to sperm fertility were not significantly different in abundance between the four highest and lowest CFI bulls, but future studies with a greater number of bulls with extremely low CFI may improve our ability to detect such metabolites when differences in means versus linear relationships are assessed.

Overall, results of this study suggest poor energy production and increased oxidative stress in bulls of subfertility. It is essential that such results are not overinterpreted due to relatively small sample size and the correlative versus causative nature of metabolite abundance with CFI. No specific metabolites identified as related to CFI in the current study were reported as significant to bull fertility by others [23,24,25]. It is valuable to note, however, that our report and each of these other pioneering studies into the sperm cell metabolome have identified that abundance of metabolites associated with pathways involving glycolysis or the TCA cycle and oxidative balance was related to bull fertility classification. Future studies that further investigate consequences of modulating sperm cell or semen levels of specific metabolites or biological pathways identified in this study and by others could further inform the scientific community of causative relationships between our findings and bull fertility.

## 5. Conclusions

Although further validation of the findings of the current study is necessary, the use of metabolites as a fertility marker to identify and remove sub-fertile bulls from a breeding population has promising future implications. Three and two metabolites had a quadratic or linear relationship with field fertility, respectively, and an additional two metabolites were more abundant in the sperm of the four lowest versus highest fertility bulls. Thus, metabolites kynurenine, xanthine, ophthalmate, tricarballylic acid and creatinine are promising fertility markers to identify subfertile bulls from a breeding population. Biological function of these metabolites indicate that subpar energy production and poor oxidative balance may partially explain reduced fertility of subfertile beef bulls. These metabolites may have future implications in the diagnosis and treatment of beef bull subfertility.

## 6. Patents

Methodology described herein is covered by US Provisional Patent Application No. 63/867,388, filed on 20 August 2025.

## Figures and Tables

**Table 1:** Fertility class, fertility index, number of A.I. services, and total motility, progressive motility, and viability of sperm of four highest and four lowest fertility Angus bulls (mean ± SEM).

Variables	High Fertility (*n* = 4)	Low Fertility (*n* = 4)	*p*-Value
Fertility Index	2.30 ± 0.35	−5.25 ± 0.93	<0.0001
Age at Collection (d)	1542.25 ± 481.36	1644.25 ± 252.13	0.86
Number of A.I. Services	4739.25 ± 1714.96	3019.50 ± 1166.17	0.45
Total Motility (%)	79.33 ± 6.87	79.37 ± 6.87	0.99
Progressive Motility (%)	62.63 ± 9.50	67.22 ± 8.95	0.64
Viability (%)	95.90 ± 1.66	92.63 ± 2.19	0.45

## Data Availability

The data presented in this study are available on request from the corresponding authors due to ongoing studies.

## Supplementary Materials

The following supporting information can be downloaded at: https://www.mdpi.com/article/10.3390/metabo16050307/s1, Table S1: Linear and quadratic relationship between all bulls and each metabolite; Table S2: Mean metabolite abundance between the four highest and four lowest fertility bulls.

## Abbreviations

The following abbreviations are used in this manuscript:

AI	Artificial Insemination
BSE	Breeding Soundness Exam
CFI	Field Fertility Index
UHPLC-HRMS	Ultra-High Performance Liquid Chromatography Coupled High Resolution Mass Spectrometry
sPLSDA	sparse Partial Least Squares Discriminant Analysis
ROS	Reactive Oxygen Species
