# Supplementary material for: Association Between Sperm Metabolites and Field Fertility in Angus Bulls
Source: Metabolites. 2026 Apr 30;16(5):307. doi: 10.3390/metabo16050307 (PMC13208153; doi:10.3390/metabo16050307)
Supplement: Supplementary file 1 [file metabolites-16-00307-s001.zip › metabolites-4224070-supplementary.pdf]

Table S1. Linear and quadratic relationship between bulls' fertility index (CFI) and each sperm metabolite.

Metabolite*	Model	Linear	Quadratic	R^2	n
3-Methylthiopropionate	0.652	0.5566	0.4367	0.0688	15
Methionine sulfoxide	0.3163	0.2519	0.177	0.17457	15
Homoserine/Threonine	0.3599	0.1879	0.1688	0.18487	13
Kynurenic acid	0.2198	0.4398	0.3448	0.2408	14
Acetyllysine	0.8184	0.6358	0.5741	0.03286	15
Alanine/Sarcosine	0.3392	0.2034	0.1667	0.17844	14
CDP-ethanolamine	0.938	0.8449	0.8338	0.01587	11
Homovanillic acid (HVA)	0.4384	0.2103	0.2111	0.12842	15
Phenyllactic acid	0.5193	0.3687	0.3102	0.1123	14
Valine/betaine	0.4873	0.2457	0.2421	0.13391	13
3-Methylphenylacetic acid	0.9248	0.9885	0.9587	0.01295	15
NADH	0.8432	0.8949	0.7768	0.03054	14
3-Phosphoglycerate	0.491	0.7603	0.5467	0.12132	14
Abscisate	0.5174	0.3718	0.3593	0.12349	13
**Kynurenine**	**0.0007**	**0.0002**	**0.0002**	**0.6986**	**15**
Pantothenate	0.7639	0.6741	0.6028	0.0478	14
1-Methyladenosine	0.7926	0.5048	0.5044	0.038	15
2-Aminoadipate	0.6018	0.5851	0.5716	0.08114	15
2-Dehydro-D-gluconate	0.5154	0.5513	0.4205	0.11354	14
AMP/dGMP	0.5085	0.3347	0.3389	0.1066	15
Aconitate	0.6936	0.5714	0.5653	0.06436	14
Adenosine	0.7025	0.4273	0.4247	0.05715	15
Allantoate	0.3694	0.3195	0.2362	0.1806	13
Aspartate	0.8632	0.9155	0.9636	0.02899	13
CMP	0.8128	0.546	0.5489	0.03396	15
Citrate/isocitrate	0.2817	0.2274	0.1616	0.19036	15
Creatine	0.6075	0.3832	0.4859	0.07972	15
**Creatinine**	**0.0503**	**0.0503**	**.**	**0.2828**	**14**
Cysteate	0.831	0.9984	0.9101	0.0331	14
D-Gluconate	0.5154	0.5518	0.4309	0.11355	15
D-Glyceraldehdye 3-phosphate	0.3658	0.8867	0.9307	0.15433	15
Deoxyinosine	0.9459	0.9423	0.9866	0.00922	15
GMP	0.8649	0.6612	0.7138	0.0239	15
Glucosamine	0.8093	0.5323	0.5334	0.03775	14
Glucose phosphate	0.9906	0.8945	0.9004	0.00171	14
Glutamate	0.7617	0.8437	0.8367	0.04435	15
Glutamine	0.8262	0.6707	0.7259	0.03132	15

Glutathione disulfide	0.764	0.9732	0.9614	0.04776	14
Glycerone phosphate	0.3669	0.8851	0.929	0.15388	15
Guanosine	0.3604	0.4802	0.313	0.15641	15
Homocysteic acid	0.4174	0.1977	0.2211	0.1355	15
Hypoxanthine	0.9	0.7398	0.7458	0.01897	14
IMP	0.8272	0.8492	0.831	0.0339	14
Inosine	0.3661	0.2489	0.1944	0.15421	15
Lactate	0.9321	0.9768	0.9117	0.0127	14
Leucine/Isoleucine	0.6766	0.4055	0.3899	0.06857	14
Methionine	0.4375	0.8059	0.521	0.13957	14
N-Acetylglucosamine	0.8815	0.6295	0.6311	0.0208	15
N-Acetylglucosamine 1/6-phosphate	0.7312	0.4508	0.4586	0.05083	15
N-Acetylglutamate	0.4902	0.3342	0.4075	0.13291	13
N-Acetylglutamine	0.3258	0.5277	0.5105	0.18448	14
N-Carbamoyl-L-aspartate	0.8153	0.5364	0.5325	0.03347	15
NAD+	0.8557	0.5848	0.5905	0.02564	15
**Ophthalmate**	**0.0578**	**0.0201**	**0.0198**	**0.4045**	**14**
Orotate	0.4697	0.2437	0.2287	0.11834	15
Phenylalanine	0.9554	0.7861	0.7719	0.00826	14
Phosphoenolpyruvate	0.3917	0.9922	0.7717	0.14462	15
Pyroglutamic acid	0.4259	0.4579	0.4089	0.15692	13
Sulfolactate	0.727	0.4375	0.4372	0.05176	15
Taurine	0.4519	0.307	0.4019	0.12398	15
Trehalose/Sucrose	0.6608	0.8603	0.8675	0.06673	15
Tryptophan	0.578	0.3924	0.3377	0.09486	14
Tyrosine	0.7903	0.6153	0.6592	0.04597	13
UDP-N-acetylglucosamine	0.7757	0.4869	0.507	0.04144	15
UDP-glucose	0.7324	0.8943	0.7511	0.05504	14
UMP	0.5184	0.3678	0.3854	0.11259	14
Uric acid	0.9267	0.9696	0.9039	0.0126	15
Uridine	0.1244	0.4091	0.198	0.31539	14
**Xanthine**	**0.0332**	**0.0333**	**0.032**	**0.5309**	**12**
Xylitol	0.9121	0.8097	0.8046	0.01659	14
aminocaproic acid	0.6577	0.3958	0.3759	0.07335	14
cAMP	0.991	0.9941	0.971	0.0015	15
phosphorylethanolamine	0.8645	0.5986	0.6077	0.02871	13
sn-Glycerol 3-phosphate	0.453	0.3561	0.5027	0.12365	15
**tricarballylic acid**	**0.0138**	**0.0138**	**.**	**0.4087**	**14**

n=number of bulls remaining in analysis after outliers removed (bulls with greater than 2.5 standard deviation from the mean were considered outliers); *bolded rows represent metabolites with model there were statistically significant

Table S2. Mean metabolite abundance between the four highest and four lowest fertility bulls for each sperm metabolite.

Metabolite *	Average Peak Area		P-Value
	High Fertility	Low Fertility	
3-Methylthiopropionate	7248.78	5011.11	0.3058
Methionine sulfoxide	46184	59841	0.4423
Homoserine/Threonine	596586	605812	0.9175
Kynurenic acid	262331	264463	0.9288
Acetyllysine	90972	82748	0.6591
Alanine/Sarcosine	685059	709267	0.8443
CDP-ethanolamine	8.0772	8.383	0.797
Homovanillic acid (HVA)	1308111	1284627	0.9332
Phenyllactic acid	140651	1414262	0.9808
Valine/betaine	979695	1103107	0.3351
3-Methylphenylacetic acid	751042	7592818	0.9199
NADH	10392	15401	0.3293
**3-Phosphoglycerate**	**1141332**	**221929**	**0.0842**
Abscisate	2837.27	1626.05	0.4097
Kynurenine	21142	17051	0.643
Pantothenate	170409	1649163	0.9063
1-Methyladenosine	256480	338098	0.5259
2-Aminoadipate	1332611	1529440	0.7623
2-Dehydro-D-gluconate	143410	1336837	0.7511
AMP/dGMP	27.4721	27.6084	0.7894
Aconitate	8674668	10464925	0.3866
Adenosine	559408	409620	0.4058
Allantoate	13742	15908	0.728
Aspartate	8654708	10145823	0.1847
CMP	672467	60632	0.7534
Citrate/isocitrate	928273	6640763	0.3536
Creatine	84427	146218	0.1839
Creatinine	33558	471745	0.1198
Cysteate	433333	423356	0.8994
D-Gluconate	606969	6345455	0.8319
D-Glyceraldehdye 3-phosphate	22.5562	21.6135	0.2147
Deoxyinosine	332536	339886	0.8996
GMP	3595240	3429523	0.9048
Glucosamine	63793	69272	0.3135
Glucose phosphate	22.561	21.6158	0.2133
Glutamate	5434374	5154726	0.8108
Glutamine	13381702	11801335	0.7273

Glutathione disulfide	110279	206051	0.3726
Glycerone phosphate	9985183	5286718	0.2929
Guanosine	436777	299911	0.4152
Homocysteic acid	176291	180705	0.9422
Hypoxanthine	71667	111713	0.321
IMP	75141	74672	0.9804
Inosine	4421945	2555420	0.2681
Lactate	31.2247	31.3669	0.7748
Leucine/Isoleucine	5906074	6803934	0.2979
Methionine	68589	90256	0.3139
N-Acetylglucosamine	246350	274874	0.789
N-Acetylglucosamine 1/6-phosphate	1206293	1151709	0.9381
**N-Acetylglutamate**	**373830**	**488745**	**0.0143**
**N-Acetylglutamine**	**17.6069**	**18.2701**	**0.0512**
N-Carbamoyl-L-aspartate	21486	24975	0.5736
NAD+	2994942	2800184	0.7736
Ophthalmate	252080	246505	0.9609
Orotate	8236856	8293574	0.983
Phenylalanine	3863452	4535621	0.3451
Phosphoenolpyruvate	80670	40843	0.1285
**Pyroglutamic acid**	**52787813**	**66427748**	**0.0602**
Sulfolactate	1771295	15020273	0.4656
Taurine	2973469	32634280	0.6063
Trehalose/Sucrose	8404281	8444089	0.9864
Tryptophan	403894	507752	0.318
Tyrosine	1255779	1595105	0.1362
UDP-N-acetylglucosamine	236912	215437	0.7757
UDP-glucose	17.6224	17.7178	0.7761
UMP	207884	156839	0.4706
Uric acid	878011	933750	0.7368
Uridine	12.9128	14.1151	0.3267
Xanthine	275437	336315	0.3801
Xylitol	636722	1518556	0.1635
aminocaproic acid	5883996	6712876	0.3536
cAMP	133040	127097	0.8548
phosphorylethanolamine	309869	324867	0.8688
sn-Glycerol 3-phosphate	7117421	5262014	0.6212
tricarballylic acid	2090976	2478886	0.1523

*Bolded rows represent metabolites with model there were statistically significant
